# Decentralizing healthcare in Norway to improve patient-centered outpatient clinic management of rheumatoid arthritis – a conceptual model

**DOI:** 10.1186/s41927-021-00215-1

**Published:** 2021-11-08

**Authors:** Alen Brkic, Jung G. Kim, Glenn Haugeberg, Andreas P. Diamantopoulos

**Affiliations:** 1 Department of Research, Sorlandet Hospital, Service Box 416, Kristiansand, Norway; 2 Kaiser Permanente Bernard J. Tyson School of Medicine, Department of Health Systems Science, Pasadena, CA USA; 3 Division of Rheumatology, Department of medicine, Sorlandet Hospital, Kristiansand, Norway; 4 Department of Rheumatology, Martina Hansens Hospital, Bærum (Oslo), Norway; 5 Division of Rheumatology, Department of Medicine, Akershus University Hospital, Kongsvinger, Norway

**Keywords:** Decision making, Chronic disease management, Telehealth, Empowerment, Rheumatology, Cost reduction, COVID, Patient-centeredness

## Abstract

A growing population of older adults and improved effective treatments for inflammatory rheumatic diseases will increase the demand for more healthcare resources that already struggle with staggering outpatient clinic waiting times. Transformative delivery care models that provide sustainable healthcare services are urgently needed to meet these challenges. In this mini-review article, a proposed Lifelong Treatment Model for a decentralized follow-up of outpatient clinic patients living with rheumatoid arthritis is presented and discussed.

Our conceptual model follows four steps for a transformative care delivery model supported by an Integrated Practice Unit; (1) Diagnosis, (2) Treatment, (3) Patient Empowered Disease Management, and (4) Telehealth. Through an Integrated Practice Unit, a multidisciplinary team could collaborate with patients with rheumatoid arthritis to facilitate high-value care that addresses most important outcomes of the patients; (1) Early Remission, (2) Decentralization, (3) Improved Quality of Life, and (4) Lifelong Sustain Remission.

The article also addresses the growing challenges for the healthcare delivery system today for patients with rheumatoid arthritis and proposes how to reduce outpatient clinic visits without compromising quality and safety.

## Background

**Fig. 1:**
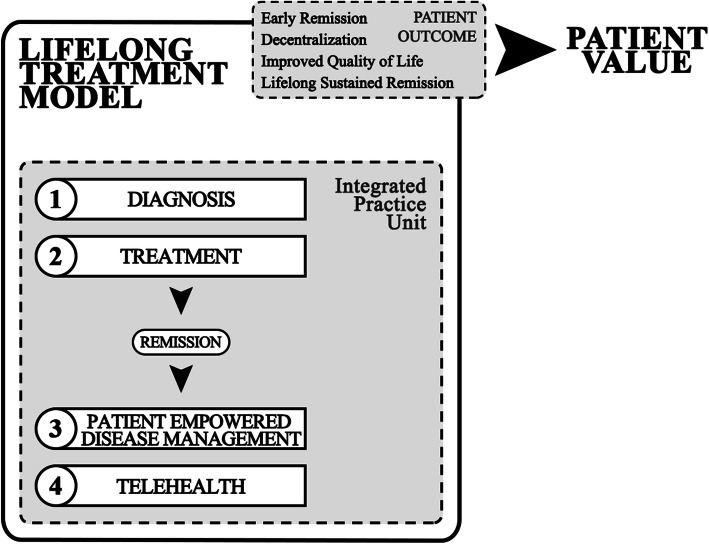
The lifelong rheumatoid arthritis patient treatment model. Legend: Not Applicable

**Fig. 2:**
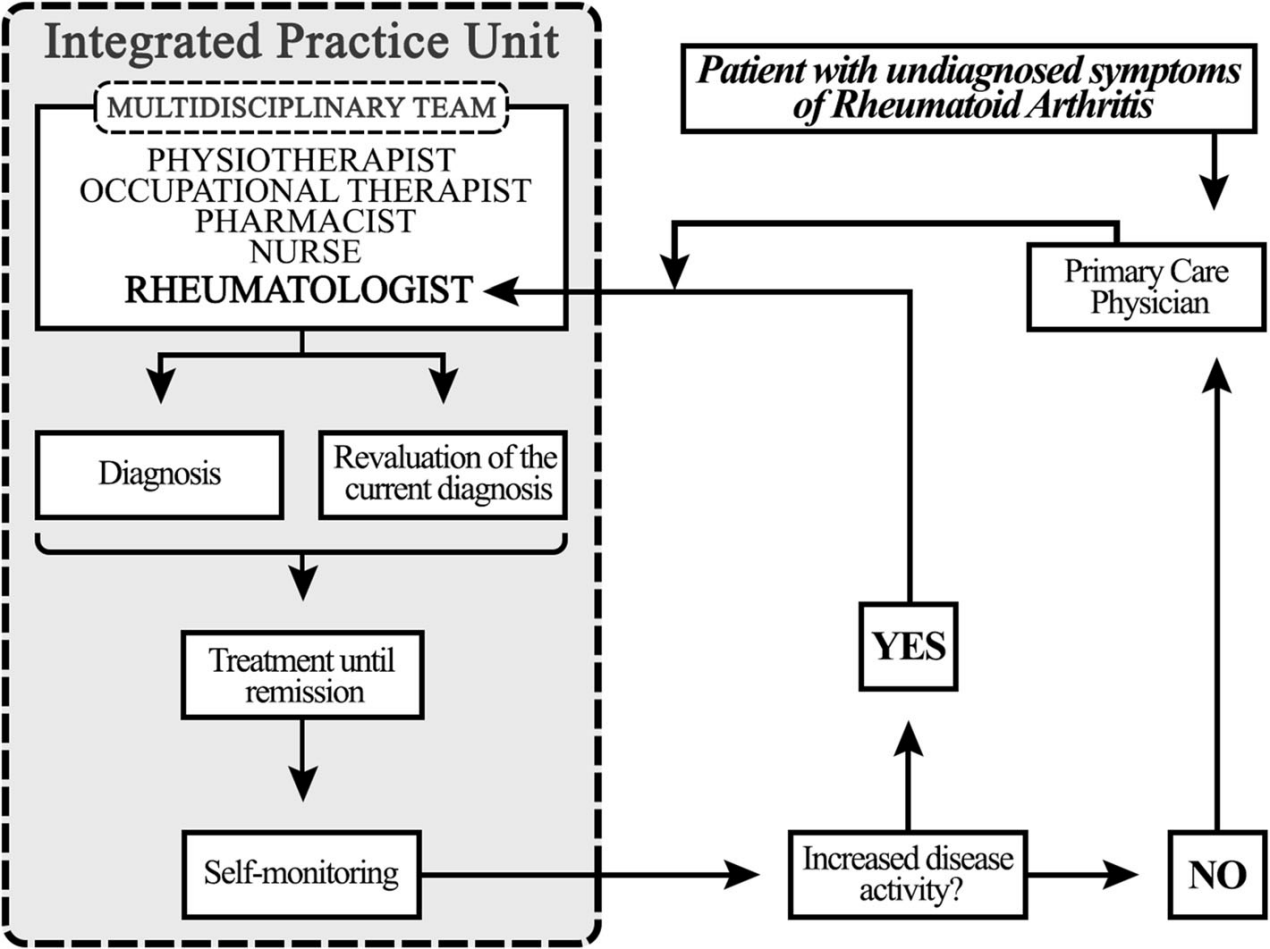
The Integrated Practice Unit (IPU) plan for patients with rheumatoid arthritis. Legend: Not Applicable

This paper proposes a “lifelong treatment model” for rheumatoid arthritis (RA) patients to meet the increasing challenges of the growing waiting list at outpatient clinics and indicate the pressure for healthcare systems to care for their patient populations. The proposed model seeks to maximize efficient use of resources, e.g., clinical visits for RA patients in remission or low disease activity, without compromising safety and quality through a decentralized follow-up and self-monitoring care model using telehealth and innovative electronic tools. Figure 1, the lifelong RA patient treatment model and Fig. 2, the Integrated Practice Unit (IPU) depicts our conceptual model and describes the interaction between the IPU, patient and primary care physicians.

Rheumatoid arthritis (RA) is a systemic autoimmune chronic inflammatory disorder primarily involving small joints of hands and feet [1]. The disease consequence for the patients includes, e.g., pain, joint stiffness, physical impairments, and fatigue that reduces the health-related quality of life (HRQoL) [2, 3]. Significant improvement in disease outcome of RA patients has been reported recently, driven by improved treatment strategies (early treatment and treating patients to target) and new drugs [4–7]. Consequently, there has been a shift in rheumatology from inpatient to outpatient clinic follow-up, thus, reducing the need for hospital beds substantially. With the reduction of hospital beds, inpatient costs have been reduced, whereas outpatient-based medication usage and costs for RA have increased. For example, in Norway from 2006 to 2018, the number of offered biological treatments increased from 7650 to 34,000 [8]. With the shift from inpatient to outpatient follow-up of patients treated with expensive and potentially harmful drugs, the number of outpatient clinic visits has increased, challenging the capacity of the healthcare system. With the growing outpatient population needing care, the wait times are increasing [9]. The COVID-19 pandemic has shown how vulnerable healthcare system resources are and highlights the need and opportunities to organize and deliver healthcare services, e.g., telehealth. Further, considering the global lockdown of healthcare systems due to the COVID-19 pandemic, the proliferation of waiting lists may be exponentially higher than in previous years [10].

Compared to nurse-led outpatient clinics, patient-centered methods have shown that implementing patient-initiated and patient-self-monitored approaches among RA patients reduces the usage of primary and secondary healthcare services without compromising patient’s clinical and psychosocial well-being [9]. As observed with patient care for epilepsy, telehealth alone improved patient-reported outcomes versus face-to-face follow-up care, and saves roughly half of the patient population from physical outpatient follow-up visits [11].

The growing number of RA patients now in remission also challenges the conventional monitoring of patients through face-to-face visits [7]. However, telehealth strategies can reduce the number of conventional face-to-face consultations without sacrificing improved disease outcomes among RA patients with low disease activity or remission [12].

Thus, there is an imminent need for a different management approach for the overall outpatient population to meet the growing challenges of the healthcare system driven by an aging population and new treatment options for RA. This need is also reflected in long-term strategies addressing the need to transform the healthcare system, including innovative patient-friendly telehealth solutions. The need for a healthcare delivery that is decentralized has been further increased in light of the COVID-19 pandemic [13–18].

In this paper, we propose a conceptual model for decentralizing the Norwegian healthcare system and optimizing patient-centeredness for RA through strategic organizational management principles. The proposal utilizes Porter’s Hierarchy of Outcome model [19] applied to the RA care setting and adjusted for administration through a decentralized approach to promote high-value care through an Integrated Practice Unit.

## Main text

### Addressing the changing rheumatoid arthritis care model in Norway

Strategic planning in the management sector has been incorporated to identify high-value methods for the end-user. In healthcare, the end-users are the patients, and the high-value methods enhance their treatment efficiency and maximize healthcare resources. Healthcare value is defined as optimizing patient outcomes proportional to the health system costs of achieving those outcomes [20]. Identifying and implementing high-value care is crucial in the rapidly changing RA treatment setting [20].

The fundamental driving component of increasing value for the patient is identifying the specific outcomes to operationalize value based on the patient’s perspective. In the case of care of RA patients in the Norwegian health system, the primary patient outcomes would focus on early remission (preventing irreversible joint damage), improved HRQoL while maintaining sustained remission throughout the patient’s life, and reducing patient dependence on hospital settings after achieving remission. These factors providing value to the RA patient and achieving optimal patient-centered management need to be systematically addressed (Fig. 1).

To achieve early RA remission, precise diagnosis and rapid evaluation are priorities. Both require a rapid-response strategy that is orientated to care for newly diagnosed or relapsing patients. Initiation of RA treatment within the first 3 months of diagnosis leads to the best possible outcomes, making the quick evaluation and treatment initiation a central feature in remission [21].

Quality of life (QoL) is the ability to enjoy life on a day-to-day basis despite the circumstances of having an illness. Increasing QoL depends on a patient’s ability to be in charge of their health and health-related environment [22–24]. While empowerment methods are related to improving QoL, not everyone is equally susceptible or autonomous in their decision-making [24–26]. Therefore, it is essential to consider the older adults and those with various degrees of cognitive impairment when assessing how to promote empowerment and implement telehealth among a patient population [27–29]. Patient-initiated and patient-controlled follow up, as well as patient-monitored treatment and laboratory tests, are all varieties of patient empowerment. Previous reports indicate that the various patient empowerment strategies effectively reduce waiting lists while maintaining disease stability without compromising the HRQoL of patients [9, 30].

When sustained remission is attained, where the patient has reached a suppressed state of disease activity or at least minimal disease activity, consistent monitoring of symptoms and therapeutically changes (due to biological side-effects and increased tolerance) is expected for a lifetime among RA patient [31]. Thus, the clinical provider is required to monitor the patient consistently. Incorporating strategies to facilitate patient-centeredness and empowerment may help leverage this increased demand and provide assurance and help patients maintain remission for RA. This strategy may require the incorporation of novel solutions like telehealth to facilitate the decentralized approach. Telehealth programs have also been implemented efficiently in the setting of the COVID-19 pandemic, where decentralization is necessary [13–18].

Reducing hospital dependency is achieved by decentralizing the decision-making away from the outpatient follow-ups at hospitals to a distant disease activity monitoring setup. This goal should also incorporate resource sustainability for a healthcare system that relies on multiple strategic decision-making levels. Decentralizing the management of rheumatology care is in its infancy [32, 33], however other fields worldwide have reported various degrees of effectiveness [34–38]. These reports emphasize that efficient processes in conjunction with effective innovation (e.g., effective treatments) work in tandem to improve outcomes.

### A proposed lifelong treatment model

#### The integrated practice unit

The chronic nature of RA requires a Lifelong Treatment Model (Fig. 1). This model commences with a patient living with undiagnosed symptoms of RA and decides to seek help at their primary care physician (PCP). The patient is referred to a rheumatologist for early diagnosis and treated until remission is achieved. The treatment management transpires with the support of the IPU (Fig. 2).

With the increasing demands on outpatient services for RA treatments, high-value care for RA patients may require delivery reforms that integrate services across the healthcare system in addition to telehealth. A multidisciplinary care team [39] (e.g., RA: rheumatologists, physiotherapists, occupational therapists, pharmacists, and nurses) would combine outpatient-based and inpatient-based systems with the overarching goal to achieve and maintain sustained disease remission in partnership with the patient. An IPU within the multidisciplinary care team is an essential component in the strategy and the intersection between the two delivery systems that operated within Norway.

The main IPU’s objective is to increase patient QoL and empowerment, improve disease outcomes, and leverage the entire healthcare team to contend with the increasing demand for clinical services. The current approach in Norway cannot achieve optimal patient value through one rheumatologist specialist in every local hospital to serve every rheumatologic patient’s needs. The IPU should incorporate features of an integrated healthcare system with enough patient volume, high-end equipment, and established clinical decision-making guidelines and thus, should be implemented in regional or tertiary hospitals to utilize the features and resources in a healthcare delivery system. Relocating the newly diagnosed RA patient through an outpatient rheumatologic clinic organized by an IPU would be particularly meaningful in efficiently serving a higher number of patients. The idea is to apply shared decision-making between the patient and their PCPs through the IPU’s guidance, model, and education. Assisting by telehealth technology, the IPU guides the RA patients to manage their disease and encourages them to self-monitor their health. At any point, if the patient struggles with symptoms that are unrelated to RA relapse, the patient can be supported by the IPU to overcome these challenges. However, if there are symptoms of increased disease activity, the patient can be referred back to the rheumatologist until the disease is once more in remission.

#### Steps 1 & 2: diagnosis and treatment

The first step of the model starts at the point of diagnosis, and according to the European League Against Rheumatism (EULAR), it should be made as soon as possible through clinical examination. Once the diagnosis is established, treatment (the second step) can be initialized. The recommended treatment strategy (also according to EULAR) bases itself on the initial examination by a rheumatologist, preferably within 6 weeks of the onset of joint swelling associated with pain and stiffness. Treatment with conventional synthetic disease-modifying antirheumatic drugs (csDMARDs) (e.g., methotrexate, unless contraindicated) should start rapidly, aiming to reach either low disease activity or remission as soon as possible (i.e., treat-to-target strategy) [21, 40]. Norway’s treat-to-target prescription guidelines dictate that RA patients only need to fail one type of csDMARDs, such as methotrexate before they can be converted to biological DMARDs (bDMARDs) [27]. As a Norwegian clinical standard, RA patients in remission using DMARDs still require direct observation of treatments and need continuous follow-up by rheumatologists from outpatient clinics or private rheumatologists outside the hospitals.

We suggest that the treatment should be introduced and monitored through the IPU until remission is achieved through validated outcome measures and patient-reported outcome measures.

#### Step 3: patient empowered disease management

Once the status of low disease activity or remission is achieved either through advanced treatments or combination therapy, the patient enters the model’s third step. The Patient Empowered Disease Management (PEDM) includes patient-centered methods such as patient-controlled care, patient-initiated care, and patient-monitored care. Each of these strategies is pivotal in reaching optimal value for RA patients.

The idea behind PEDM is to have the patient govern their illness while acquiring control and mastery in the process. Regaining control over their health is an empowerment method that eventually can lead to a better quality of life and well-being. Furthermore, PEDM may relieve the healthcare providers (HCP) from taking care of the patient throughout their sustained remission (i.e., throughout their life), since through this method, the patient can be given the trust to handle their disease. Indirectly, the PEDM builds a shared responsibility platform between the patients and the HCPs that addresses how to achieve the patient’s optimal values together. In advance, the IPU can provide guidance and educate patients to manage their disease and request a follow-up visit when needed. Shared responsibility, self-reporting, and external observation measuring are considering pivotal factors in patient-centered management [41, 42]. Therefore, patients should learn to recognize a disease flare or severe side effects while continuing their treatment. They should also understand their laboratory test better and monitor their disease by using telehealth devices. The PCPs should also be educated using PEDM with the patient and contact the IPU with the patient if any disease status changes occur.

When patients understand the factors affecting their health, they can feel empowered and experience an enhanced quality of life. Having autonomy, knowledge, and control to make these decisions may create stronger self-esteem, better self-affirmation, and directly improve their well-being [24].

#### Step 4: maintenance through telehealth

As the final step, while in remission, the informed PEDM-patient should be followed up with telehealth monitoring by rheumatology-specialized nurses or artificial intelligence (AI) algorithms [43, 44]. These qualified nurses or algorithms intend to collaborate with primary healthcare services. Using telehealth technology, a strategy to facilitate follow-up care and ensure that patients with deteriorating diseases can receive appropriate and rapid therapy can be implemented. This strategy can be further enhanced by AI-led software (e.g., chatbots) [43, 44].

Implementing follow-up methods based on prescheduled visits at outpatient clinics in various other chronic inflammatory diseases (e.g., inflammatory bowel diseases) where remission is attainable doubtfully suits with the unpredictable clinical course of the disease [45]. Prescheduled visits can compromise the quality of care and accessibility for the sickest while inefficiently using resources within a healthcare system. However, monitoring these patients through telehealth and implementing self-initiated follow-up could add value to the healthcare systems by improving efficiency and reducing costs without compromising disease outcomes [9, 20, 45]. The reduced utilization of healthcare at the specialist level may release resources allocated to non-responders and provide rapid evaluation for newly diagnosed patients or patients with a disease flare-up [45]. Patients with direct access to rheumatology outpatient clinics resulted in 38% fewer hospital appointments and better satisfaction and confidence in the system [30].

Patient self-monitoring is a well-accepted and common practice among other chronic diseases (e.g., diabetes, hypertension, and congestive heart failure), supporting decentralized care strategies while improving patient outcomes [46]. Telehealth tools would further facilitate self-monitoring and remote patient surveillance. A double-blind, multicenter, and randomized controlled study with 12 months follow-up showed that monitoring inflammatory bowel disease patients via a telehealth system was safe and reduced outpatient visits and hospital admissions compared with standard care [45].

In tandem, another study found that up to 70% of patients with RA were reported not to adhere to their prescribed medication [47]. Therefore, telehealth tools may also improve medication compliance by reminding patients to take their prescribed treatments [48].

The use of innovative telehealth tools has also been recommended for close monitoring and follow-up of patients with inflammatory arthritis [49]. For inflammatory arthritis, various remote monitoring tools are in use or being developed with the potential to help improve disease management [49]. Patient-oriented telehealth systems for remote patient monitoring should meet the following: 1. The patient’s information on telehealth platforms should be available as reports and on time-view graphs for both patients and HCPs 2. The system should improve drug compliance by reminding patients to take their medication. 3. The system should make it easy for the patient to communicate with the outpatient clinic. 4. The system should also facilitate the self-administered booking of visits at the outpatient clinic or the private specialist (e.g., if disease status is deteriorating). 5. The knowledge regarding their disease should be easily accessible at the patients’ convenience, including information about symptoms, medication and their side effects, and laboratory values related to disease progression. A randomized study examining RA patients using telehealth monitoring in this manner is currently underway. It aims to evaluate disease outcomes and the health-related cost using patient-centeredness through telehealth [50]. Other studies showed a positive effect from using telehealth monitoring of COVID-19 patients [51]. Incorporating telehealth monitoring in COVID-19 patients with an effective managerial strategy may provide early detection of declining health status and efficient use of healthcare resources, and may reduce the need for physical contact with HCPs and subsequently reduce the potential risk of infection [52]. Currently, Norway has an ongoing project to use telehealth monitoring on COVID-19 patients isolated at homes and within the hospital. Implementing inpatient-based telehealth on COVID-19 infected patients reduces the infection risk of the HCPs and saves personal protective equipment [53].

## Conclusion

The significant progress in improving RA patient outcomes is the synergy of new treatments and novel therapy strategies coupled with vigilant disease activity monitoring from early diagnosis. With the increased demand for outpatient clinics, the unintended consequences include long waiting lists. This will reduce access to the specialist healthcare system for patients with RA flare-ups, medication side-effects, or new-onset disease.

A decentralized strategy at a national level that addresses routine care to prevent care fragmentation and inefficiencies may be required to mitigate the growing bottle-neck effect for access to outpatient clinics. Implementing the lifelong proposed model focusing on telehealth can maximize high-value care for RA patients by achieving optimal health outcomes at the lowest cost and greater efficiency for the healthcare system. Such a model can be implemented in other settings caring for COVID-19 patients. A corresponding pandemic-IPU can be applied around the COVID-19 patients, where their values are equally addressed in the same manner described above. The model’s success may depend on the healthcare system to prioritize the patient’s definition of health outcomes in the face of constraints. While our proposed model is illustrated within the Norwegian healthcare system, its applicability to other countries is feasible provided that the objective is to mobilize innovations across existing healthcare systems that could benefit from improved workflow efficiencies.

Amid a growing aging population, advanced RA treatments, yet restricted admissions at hospitals, utilizing a high value-based approach instead of a supply-driven system will be mandatory to sustain healthcare systems. A transformative care delivery model supported by an Integrated Practice Unit could be one approach to decentralize health systems and promote high-value care that matters most to RA patients.

## Data Availability

Not applicable.

## Abbreviations

AI: Artificial Intelligence
bDMARD: Biological disease-modifying antirheumatic drugs
csDMARD: Conventional synthetic DMARD
EULAR: European League Against Rheumatism
HCP: Healthcare provider
HRQoL: Health-Related Quality of Life
IPU: Integrated Practice Unit
PEDM: Patient Empowered Disease Management
PCP: Primary Care Physician
QoL: Quality of Life
RA: Rheumatoid arthritis
